# Commentary: Surveillance of Crimean-Congo Haemorrhagic Fever in Pakistan

**DOI:** 10.3389/fpubh.2017.00132

**Published:** 2017-06-06

**Authors:** Tauqeer Hussain Mallhi, Yusra Habib Khan, Nida Tanveer, Amer Hayat Khan, Muhammad Imran Qadir

**Affiliations:** ^1^Discipline of Clinical Pharmacy, School of Pharmaceutical Sciences, University Sains Malaysia, Penang, Malaysia; ^2^Institute of Molecular Biology and Biotechnology, Bahauddin Zakariya University, Multan, Pakistan; ^3^Punjab Medical College, Faisalabad, Pakistan

**Keywords:** vector borne diseases, Crimean-Congo hemorrhagic fever, Pakistan, climate change, epidemics, surveillance

Karim et al. (1) in their recent correspondence in “*Lancet Infectious Diseases*” reported nationwide distribution of Crimean-Congo hemorrhagic fever (CCHF) in Pakistan with the highest prevalence recorded in Balochistan province (*n* = 38/86). Authors have discussed that unchecked transportation of animals from Iran and Afghanistan to Balochistan is a primary contributing factor to the highest prevalence of positive cases in the province and urged the enforcement of border control regulations (1). We agree that substantial reduction in CCHF cases in Balochistan can be observed with appropriate monitoring and health checks on cross-border movements. However, we felt inclined to share our point of view about the recent increase in CCHF cases in Balochistan. We believe that there are some more important factors that primarily explain the recent surge in CCHF in Balochistan and must be addressed by the Government of Pakistan to mitigate the disease burden.

Balochistan province is situated in the arid mountainous south-west quadrant of Pakistan and accounts about 45% of total area of Pakistan with clustered population that is predominantly rural in distribution (2). It is a highly neglected province of Pakistan, and most of its population does not have access to vector control vaccines and other health-care facilities (3, 4). Prevalence data indicate that most of the positive cases were observed in rural areas of the province where animal husbandry is the major source of income (5). These rural dwellers have low literacy rate and are unaware of tick borne viruses. They live alongside their cattle without any preventive measures and not only consume milk and meat to survive but also use their dungs for wound healing (6, 7). All these practices compound significantly on the populations susceptibility to CCHF. Moreover, transportation of these animals to urban areas for business purposes may cause rural to urban viral spillover. Such transportations increase during Eid-ul-Adha festival, an annual religious festival observed by Muslims, where millions of animals are sacrificed in Pakistan, thereby increasing the human–animal contact. Pakistan has experienced various nosocomial outbreaks of CCHF, and Eid-Ul-Adha is regarded as a vulnerable period for these outbreaks (8).

Another factor that is believed to be an important perpetrator of high disease susceptibility, and transmission is nomadic lifestyle, traveling of people from place to place in search of fresh pasture for their livestock. Nomads do not possess permanent abode, resides along the banks of the river and move as the weather and water supply shifts. Nomadic tribes are present in the Balochistan (more than 60% of province population) regions and shift from one place to the other to fulfill their needs (6, 7, 9). These tribes along with their animals have potential to transmit disease to non-endemic regions. CCHF outbreaks typically occur following the migration of nomadic people and livestock (10), therefore we urged the national authorities to keep vigil on such migratory activities through appropriate monitoring systems. Moreover, the rise in temperature over the past few years is more pronounced in Balochistan as compared to the other provinces (11), and we believe that climate change and dry spells also equally contribute to the outburst of CCHF in Balochistan. In addition, deplorable health-care system in both rural and urban Balochistan is causing difficulties to prevent infectious diseases in the Province (3). Taken together, rural dwellers and nomads are not only at increased risk of contracting CCHF virus but are also significant predisposing factors of disease spillover. Provision of education and appropriate health facilities to these people is of paramount importance to combating CCHF in Pakistan. Last but not least, there is a dire need to introduce urgent and aggressive reforms to improve health-care system of Balochistan.

## Author Contributions

All the authors equally contributed to the manuscript. YK, TM, and AK searched for literature to support main content of the manuscript. TM and YK drafted the manuscript. NT and MQ revised manuscript and gave final approval to submit this manuscript. However, all the authors are agreeing to submission of this manuscript.

## Conflict of Interest Statement

The authors declare that the research was conducted in the absence of any commercial or financial relationships that could be construed as a potential conflict of interest.
